# The SCIDOTS Project: Evidence of benefits of an integrated tobacco cessation intervention in tuberculosis care on treatment outcomes

**DOI:** 10.1186/1747-597X-6-26

**Published:** 2011-09-23

**Authors:** Ahmed Awaisu, Mohamad Haniki Nik Mohamed, Noorliza Mohamad Noordin, Noorizan Abd Aziz, Syed Azhar Syed Sulaiman, Abdul Razak Muttalif, Aziah Ahmad Mahayiddin

**Affiliations:** 1 Clinical Pharmacy and Practice Section, College of Pharmacy, Qatar University, P.O. Box 2713, Doha, Qatar; 2 Department of Pharmacy Practice, Kulliyyah of Pharmacy, International Islamic University Malaysia, 25200 Kuantan, Pahang, Malaysia; 3 Department of Health Economics & Finance, Institute for Health Management, NIH, Ministry of Health, 59000 Kuala Lumpur, Malaysia; 4 Faculty of Pharmacy, Universiti Technology Mara, 5600 Puncak Alam, Malaysia; 5 Department of Clinical Pharmacy, School of Pharmaceutical Sciences, Universiti Sains Malaysia, 11800 Penang, Malaysia; 6 Department of Respiratory Medicine, Penang Hospital, 10990 Penang, Malaysia; 7 Institut Perubatan Respiratori, 53000 Wilayah Persekutuan Kuala Lumpur, Malaysia

## Abstract

**Background:**

There is substantial evidence to support the association between tuberculosis (TB) and tobacco smoking and that the smoking-related immunological abnormalities in TB are reversible within six weeks of cessation. Therefore, connecting TB and tobacco cessation interventions may produce significant benefits and positively impact TB treatment outcomes. However, no study has extensively documented the evidence of benefits of such integration. SCIDOTS Project is a study from the context of a developing nation aimed to determine this.

**Methods:**

An integrated TB-tobacco intervention was provided by trained TB directly observed therapy short-course (DOTS) providers at five chest clinics in Malaysia. The study was a prospective non-randomized controlled intervention using quasi-experimental design. Using Transtheoretical Model approach, 120 eligible participants who were current smokers at the time of TB diagnosis were assigned to either of two treatment groups: conventional TB DOTS plus smoking cessation intervention (integrated intervention or SCIDOTS group) or conventional TB DOTS alone (comparison or DOTS group). At baseline, newly diagnosed TB patients considering quitting smoking within the next 30 days were placed in the integrated intervention group, while those who were contemplating quitting were assigned to the comparison group. Eleven sessions of individualized cognitive behavioral therapy with or without nicotine replacement therapy were provided to each participant in the integrated intervention group. The impacts of the novel approach on biochemically validated smoking cessation and TB treatment outcomes were measured periodically as appropriate.

**Results:**

A linear effect on both 7-day point prevalence abstinence and continuous abstinence was observed over time in the intervention group. At the end of 6 months, patients who received the integrated intervention had significantly higher rate of success in quitting smoking when compared with those who received the conventional TB treatment alone (77.5% vs. 8.7%; p < 0.001). Furthermore, at the end of TB treatment (6 months or later), there were significantly higher rates of treatment default (15.2% vs. 2.5%; p = 0.019) and treatment failure (6.5% vs. 0%; p = 0.019) in the DOTS group than in the SCIDOTS group.

**Conclusion:**

This study provides evidence that connecting TB-tobacco treatment strategy is significant among TB patients who are smokers. The findings suggest that the integrated approach may be beneficial and confer advantages on short-term outcomes and possibly on future lung health of TB patients who quit smoking. This study may have important implications on health policy and clinical practice related to TB management among tobacco users.

## Background

Tuberculosis (TB) and tobacco smoking are currently two formidable public health concerns and independently pose a considerable threat to global health [1-3]. The current estimates put the annual global mortality from the two epidemics at over six million [2,3]. It is remarkable to note that TB and tobacco use are co-prevalent in many developing nations and these nations are said to be doubly burdened by the collision of the two epidemics [1,4]. In addition, there is substantial and overwhelming evidence to conclude that smoking is strongly linked to TB disease and leads to poor treatment outcomes [5-11]. It is consistently reported also that the prevalence of smoking among TB patients was high when compared with non-TB controls or the general population [12-17]. Consequently, this subject has in recent years attracted and received a widespread attention among the scientific community around the world.

Although there is paucity of data on the direct effects of smoking cessation on TB treatment outcomes [18], studies suggest that smokers are at an increased risk of treatment default, treatment failure, and relapse after successful treatment [8-11,19]. It is also well documented that tobacco smoking suppresses both cell- and humoral-mediated immunity thereby causing TB infection and worsening the outcomes of treatment [20]. However, most of the immunological abnormalities induced by smoking including decreased level of circulating immunoglobulins, decreased ratio of CD4^+ ^to CD8^+ ^lymphoctyes, and decreased release of proinflammatory cytokines are reversible within six weeks after smoking cessation [20-23]. Thus, it is strongly recommended that smoking cessation using both cognitive behavioral therapy and pharmacotherapeutic approach be incorporated as a standard of care in directly observed therapy short-course (DOTS) and other TB treatment strategies [14,18].

Furthermore, a few guidelines and educational series addressing the control of tobacco use in TB settings have recently been developed [24-32]. Despite the overwhelming evidence of association between TB and tobacco use, the recent availability of guidelines on how to integrate the management of these related epidemics, and the anticipated benefits of quitting smoking, there are several unanswered questions which warrant further research [4,14]. Connecting the two healthcare interventions could produce significant benefits and is apparently logical [4,14]. What are the potential benefits of the integrated approach in improving the outcomes of TB among smokers? In order to address this query, research efforts are needed to determine both the short-term and long-term benefits of smoking cessation in TB care.

The results of such studies could guide the development of evidence-informed practice guidelines, devoid of mere speculations and assumptions. In an effort to establish an evidence and policy direction and to expand the current knowledge of the impact of integrated TB-tobacco treatment strategy in improving treatment outcomes, quality of life, and reducing healthcare resources utilization, we set out to conduct intervention studies related to above in Malaysia. The present study, known as the SCIDOTS Project, was primarily aimed to evaluate the impact of adding smoking cessation intervention (SCI) to conventional DOTS for TB on tobacco abstinence rates and TB treatment outcomes.

## Methods

### Study Design

We conducted a prospective non-randomized controlled intervention study using quasi-experimental design. New cases of TB who currently smoke cigarettes at the time of diagnosis were assigned to either of two intervention groups, based upon their readiness to quit smoking measured through Transtheoretical Model approach [33]: conventional TB DOTS alone (comparison or DOTS group) or the conventional TB DOTS plus SCI (integrated intervention or SCIDOTS group).

The non-equivalent comparison group design was used instead of a randomized controlled trial (RCT) design to overcome practical issues and ethical imperatives associated with the RCT among TB patients who smoke.

### Study Setting and Ethical Approval

A multi-centered study was conducted at five respiratory clinics: four located within Penang State health districts and one in Wilayah Persekutuan Kuala Lumpur, Malaysia. The selected clinics were considered as TB referral centers from which all diagnosis or confirmation of TB cases must come from among the population they serve. Initiation of treatment for confirmed TB cases must also stem from such centers. The study was carried-out during the period June 2008 to December 2009. The study was approved by the Medical Research Ethics Committee of the National Institutes of Health, Ministry of Health, Malaysia.

### Study Sample and Participants

The sample of the study comprised of all current manufactured cigarette smokers newly diagnosed with active TB. Patients were identified based on their self-reported tobacco use during TB diagnosis. They were then referred at the point of initiating DOTS regimen by the attending physician to TB DOTS providers who were specially trained to provide SCI in TB setting [34]. Patients were recruited into the study upon fulfillment of eligibility criteria and written informed consent.

The primary analysis on which sample size requirements were based is the comparison of the proportion of patients cured of TB (cure rates) and treatment failure rates between the two treatment groups. For the primary outcome comparison between the integrated intervention (SCIDOTS) group and the comparison (DOTS) group, a statistical power (1- β) of 80% to detect a moderate effect size at a 95% confidence level was set. Since DOTS has a target to achieve a 95% cure rate in previous reports, we assumed a cure of 95% in the intervention arm (p_1 _= 0.95) and 75% in the control arm (p_2 _= 0.75). A minimum sample size of 48 subjects was estimated per treatment arm for the study to have a statistical power of 80% to detect a difference of 20% in TB cure rates with a 2-sided alpha level of 0.05. Assuming a dropout rate of 25%, about 60 participants would be required for each treatment arm. Therefore, a total of 120 subjects (60 for each treatment group) was targeted in the study.

### Enrollment Procedures and Eligibility Criteria

The 5A's strategy (Ask, Advise, Assess, Assist, Arrange) and the Transtheoretical Model of Stages of Change were used in the assignment of patients into the two treatment groups of the study [33,35]. First, all newly diagnosed TB patients were "asked" about their smoking status. Patients who declared their smoking status as current smokers were strongly and clearly "advised" to quit smoking, given the benefits of cessation and linking it with their current disease status. Patient's willingness to quit smoking was "assessed" using the 5-stage Transtheoretical Model (precontemplation, contemplation, preparation, action, and maintenance) and motivation assessment. Only patients in the preparation stage (defined as willingness to quit smoking within the next 30 days), or contemplation stage (considering quitting in the next 6 months, but not in the next 30 days), or precontemplation stage (not thinking about changing in the next 6 months) were invited to participate in the study by individual letters served during initiation of DOTS. Sixty patients motivated to quit smoking (in preparation stage) who gave their consent were enrolled into the integrated intervention group (to receive SCI in addition to DOTS), whereas another 60 unmotivated patients (in precontemplation and contemplation stages) willing to participate in the research were recruited into the usual care group (to receive conventional DOTS regimen).

Patients were invited to participate in the study if they fulfilled the following criteria: (1) current manufactured cigarette smokers diagnosed with active pulmonary TB: either sputum smear-positive or smear-negative, based on classification from Malaysian or WHO treatment guidelines, [36,37], (2) classified under treatment Category I (new TB cases), (3) patients of both sexes, aged 18 years and above, and (4) patients in preparation, precontemplation, or contemplation stages of behavior change. Furthermore, newly diagnosed TB patients were excluded from the study if: (1) they were in action and maintenance stages of behavior change, (2) have extrapulmonary TB only (involving CNS, pericardium, adrenal etc), (3) they have multi-drug resistant tuberculosis at diagnosis, (4) they are living with or newly diagnosed with HIV/AIDS, (5) they are classified as Category II (relapse, treatment failure, and treatment after default) or Category III (chronic TB), and (6) they fulfilled the eligibility criteria, but unwilling to participate in the study or unable to understand the contents of the informed consent form.

**Figure 1:**
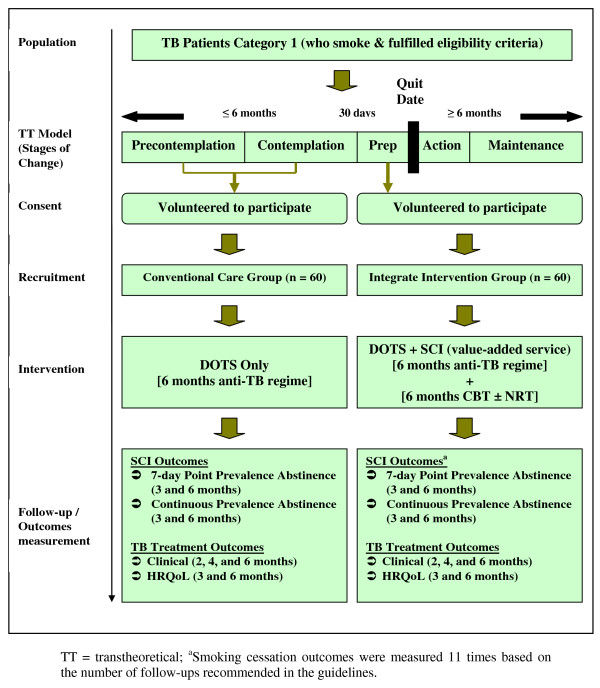
**Flowchart of subjects' enrollment/allocation and study methodology**.

Figure 1 schematically illustrates the allocation of study participants into groups and the methods used in the study.

### Interventions and Monitoring

At the first visit, patients in the intervention group received an overview of the integrated DOTS plus SCI program, whereas the usual care group received an overview of DOTS plus conventional TB counseling. Questionnaires to assess tobacco use and smoking history were completed and all patients took the Fagerström test for nicotine dependence (FTND) at baseline. During this visit, patients in the intervention group were asked to establish a target quit date (within one month from the first visit).

Patient-centered intervention techniques were employed for all recruited patients, using the 5A's strategy. At the initial visit (i.e. on the quit date) and each subsequent visit, patients in the intervention group received personalized behavioral counseling, educational materials, and refills of drug prescriptions related to smoking cessation; in addition to DOTS for TB. The behavioral counseling was individualized to work with patients in modifying behaviors and achieving success. Drug therapy for tobacco cessation comprised of four types of nicotine replacement therapy (NRT) products (nicotine gum 2 mg and 4 mg, nicotine transdermal patch, and nicotine inhaler).

Patients in both treatment groups went to the chest clinic on daily-basis to receive DOTS. Follow-up schedules for TB were on 2-monthly basis (baseline, end of second, fourth, and sixth month). The follow-up clinic appointments for SCI (beginning on the quit date) were as follows: weekly for the first month, fortnightly for the second and third month, and monthly from the fourth to the sixth month; giving rise to a total of 11 follow-up visits. NRT and behavioral counseling were modified throughout the follow-up period after evaluation of the patient's progress. Validation of cessation was performed via breath carbon monoxide (CO) monitoring using Micro™-Smokerlyzer™, one-way valve single-use mouthpieces, suitable for infection patients (Bedfont Scientific Ltd & decode, UK) and saliva cotinine dip-stick test (NikAlert^®^). Patient-reported nicotine withdrawal symptoms were measured using Wisconsin Smoking Withdrawal Scale (WSWS), validated in Malay language.

Participants were monitored during each follow-up visit for nicotine dependence, exhaled CO level, and nicotine withdrawal symptoms. Further, saliva cotinine testing was conducted during the last follow-up visit as a biochemical measure of verifying quitting. Both CO and cotinine are widely used as biomarkers for confirming smoking abstinence. For each participant in the intervention group, these measurements were conducted 12 times including baseline. Each subject was followed for a period of at least 6 months to determine the outcomes of TB and tobacco dependence treatment.

### Statistical Analysis

All data were double-entered into SPSS version 16.0 software package (SPSS Inc., Chicago, IL) by one researcher and two research assistants using two separate databases that were later compared and differences reconciled by re-checking the raw data. Patients' demographic information, baseline TB- and tobacco-related characteristics were calculated as mean ± SD for continuous variables and as proportions for categorical variables. To explore the comparability of demographic, TB- and smoking-related characteristics between the two treatment groups, homogeneity of the baseline data was measured using two-sample tests including Student's *t*-test for normally distributed continuous variables, and Pearson's chi-square (χ^2^) or Fisher's exact test for categorical variables. TB treatment outcomes were compared between the integrated intervention and the comparison groups using Pearson's χ^2 ^or Fisher's exact test and Mann Whitney-U test at 2 months, 4 months, and 6 months or end of treatment. These nonparametric statistical tests were also applied to compare smoking abstinence rates (continuous abstinence and point prevalence abstinence) between the two treatment arms at 3 months and 6 months.

## Results

Of the 120 eligible patients recruited into the study, 86 patients (71.7%) completed the intervention and follow-up; 40 participants (46.5%) received the SCIDOTS, whereas 46 participants (53.5%) received the conventional DOTS alone. All the 86 patients were included in the data analysis. Thirty-four of the enrolled participants were lost to follow-up, giving rise to an overall attrition rate of 28.3%. During the intervention and follow-up period, 20 (33.3%) and 14 (23.3%) participants in the integrated intervention and the comparison arms respectively were lost to follow-up. In the intervention group, 10 patients withdrew their consent, three were diagnosed as HIV-positive, six were either transferred to other treatment centers or unable to contact, and one was incarcerated. In addition, nine patients in the comparison group withdrew their consent, one turned out to be HIV-positive, whereas four were either transferred to other treatment centers or not contactable. Consequently, data were collected from 40 patients and 46 patients in the SCIDOTS and the DOTS groups respectively throughout the study period of six months or longer.

### Group Equivalence for Demographic, Smoking and Disease-Related Characteristics

We compared the study groups in order to ascertain whether they were similar in related characteristics, despite lack of randomization. Subjects in the two groups were homogenous with respect to most of the characteristics. Table 1 presents the comparison of the demographic characteristics of the study participants. The groups did not differ significantly with regards to the distribution of age (t =-1.33, p = 0.186), gender (Fisher's exact p = 1.000), ethnicity (Fisher's exact p = 0.431), and marital status (Fisher's exact p = 0.961).

**Table 1:** Demographic characteristics of the study participants (N = 86)

Characteristic	Comparison Group (DOTS only) (n = 46)	Intervention Group (DOTS + SCI) (n = 40)	p-value^a^
Mean age at time of enrolment (years)	45.78 ± 14.42	41.62 ± 14.41	0.186^b^
Mean body weight (kg)	50.33 ± 8.82	53.30 ± 10.67	0.162^b^
Male gender, no. (%)	45 (97.8%)	40 (100.0%)	1.000
Ethnicity, no. (%)			0.431
• Malay	29 (63.0%)	30 (75.0%)	
• Chinese	11 (23.9%)	8 (20.0%)	
• Indian	5 (10.9%)	1 (2.5%)	
• Other	1 (2.2%)	1 (2.5%)	
Marital status, no. (%)			0.961
• Single	17 (37.0%)	14 (35.0%)	
• Married	24 (52.2%)	22 (55.0%)	
• Divorced	2 (4.3%)	1 (2.5%)	
• Other	3 (6.5%)	3 (7.5%)	

^a^Fisher's exact test and ^b^Independent t-test were applied in calculating p-values.

DOTS = directly observed therapy short-course; SCI = smoking cessation intervention

In addition, most of the baseline smoking habits and smoking-related characteristics of the enrolled TB smokers were not significantly different between the two treatment groups (Table 2). In both groups, TB patients smoked about 16 cigarettes per day on the average. Patients in the DOTS group had smoked for 28.63 ± 13.25 years in their lifetime vs. 24.80 ± 13.49 years for those in the SCIDOTS group (t = -1.326, p = 0.188). However, the distribution of nicotine dependence significantly differed between the two groups and subjects in the DOTS group were more dependent on nicotine than those in the SCIDOTS group (FTND, 5.43 ± 1.96 vs. 4.32 ± 2.26; t = -2.439, p = 0.017). Moreover, the mean CO level of the DOTS group was higher when compared with the SCIDOTS group (7.61 ± 3.61 ppm vs. 5.93 ± 2.83 ppm; t = -2.370, p = 0.020). The clinical presentations of TB were equally distributed between the treatment groups during diagnosis. Nearly all patients (97.8-100%) in both groups presented with cough of more than two weeks duration, but hemoptysis and dyspnea were relatively less common. Similarly, tuberculin skin test (TST) and chest X-ray findings were similar among the DOTS and the SCIDOTS groups.

**Table 2:** Smoking history and baseline smoking-related characteristics of the study participants (N = 86)

Characteristic	Comparison Group (DOTS only) (n = 46)	Intervention Group (DOTS + SCI) (n = 40)	p-value^a^
Smoking habits/history			
• Mean age when began smoking (years)	17.37 ± 3.55	16.88 ± 4.31	0.561
• Mean duration of smoking (years)	28.63 ± 13.25	24.80 ± 13.49	0.188
• Mean cigarettes per day	16.70 ± 9.62	16.02 ± 8.54	0.735
Mean FTND score	5.43 ± 1.96	4.32 ± 2.26	0.017
Nicotine dependence at enrolment, no. (%)			0.048^b^
• High (7-10)	15 (32.6%)	8 (20.0%)	
• Moderate (4-6)	25 (54.3%)	18 (45.0%)	
• Minimal (less than 4)	6 (13.0%)	14 (35.0%)	
Previous attempt to quit, no. (%)	18 (39.1%)	21 (52.5%)	0.214^b^
Mean breath CO (ppm)	7.61 ± 3.61	5.93 ± 2.83	0.020
Mean WSWS score	53.09 ± 10.69	56.00 ± 12.42	0.246

^a^Independent t-test was applied in calculating p-values; ^b^Chi-square (χ^2^) test.

DOTS = directly observed therapy short-course; FTND = Fagerström test for nicotine dependence; SCI = smoking cessation intervention; WSWS = Wisconsin smoking withdrawal scale

### Smoking Cessation Interventions and Outcomes Monitoring

All participants enrolled in the intervention group were provided with either CBT alone (40%) or combination of CBT and NRT products (60%). Of those who received concomitant therapy, about one-third were on nicotine gum 2 mg. Only 8.3% of the integrated intervention cohort had received therapy with nicotine gum 4 mg and nicotine inhaler each. Table 3 provides additional details on the types of SCI provided and the NRT products used.

**Table 3:** Smoking cessation intervention for the integrated intervention group (n = 40)

Intervention	No. (%)
**Type of smoking cessation intervention**	
CBT only	16 (40.0%)
CBT + NRT	24 (60.0%)
**Pharmacotherapeutic agent (NRT product type)**	
Nicotine gum 2 mg	8 (33.3%)
Nicotine gum 4 mg	2 (8.3%)
Nicotine patch (5 mg/10 mg/15 mg)	2 (8.3%)
Nicotine inhaler 10 mg	3 (12.5%)
Nicotine gum 4 mg/Nicotine gum 2mg	3 (12.5%)
Nicotine gum 2 mg/Nicotine patch	1 (4.2%)
Other combinations	5 (20.8%)
**Frequency of CBT encounters**	
9	1 (2.5%)
10	2 (5.0%)
11	36 (90.0%)
12	1 (2.5%)

CBT = Cognitive behavioral therapy; NRT = Nicotine replacement therapy

Both expired CO level and FTND were consistently and significantly lower in the SCIDOTS group when compared with the DOTS group for all time periods (Table 4). When tested for cotinine using saliva as a biological matrix at the end of the treatment, 77.5% vs. 17.4% of patients in the SCIDOTS and the DOTS groups respectively had concentration of 0-10 ng/mL (negative result). Refer to Table 4 for more details on these.

**Table 4:** Smoking cessation monitoring according to group assignment*

Monitoring parameter	DOTS (n = 46)	SCIDOTS (n = 40)	**p-value**^†^
**Baseline (Week 0)**			
Breath CO (ppm)	7.61 ± 3.61	5.93 ± 2.83	0.020
FTND	5.43 ± 1.96	4.32 ± 2.26	0.017
WSWS	53.09 ± 10.69	56.00 ± 12.42	0.246
**End of 3^rd ^month post quit date (Week 12)**			
Breath CO (ppm)	7.30 ± 2.99	1.98 ± 1.25	< 0.001
FTND	4.87 ± 2.10	0.15 ± 0.53	< 0.001
WSWS	51.39 ± 9.81	47.95 ± 10.70	0.123
**End of 6^th ^month post quit date (Week 24)**			
Breath CO (ppm)	8.00 ± 3.58	1.83 ± 2.47	< 0.001
FTND	4.57 ± 1.70	0.25 ± 0.71	< 0.001
WSWS	51.87 ± 9.43	48.20 ± 9.14	0.072
Negative cotinine test (0-10 ng/mL), n (%)	8 (17.4%)	31 (77.5%)	< 0.001^‡^

* Plus-minus values are means ± SD; ^†^p-values were calculated with the use of independent t-test for all continuous data reporting mean values; ^‡^p-value was calculated using Chi-square (χ^2^) test.

FTND = Fagerström test for nicotine dependence; WSWS = Wisconsin smoking withdrawal scale

### Effect of the Intervention on Smoking Cessation Outcomes

**Figure 2:**
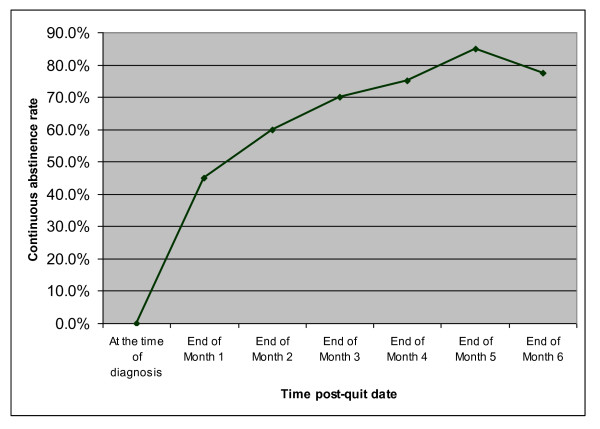
**Linearity of smoking cessation outcome for the intervention group: Continuous abstinence (CA)**.

A linear effect on biochemically-confirmed 7-day point prevalence abstinence (PPA) and continuous abstinence (CA) was observed over time in the intervention group; both the 7-day PPA and the CA rates were 45.0% (18/40) at one month after the quit date, but increased to 62.5% (25/40) and 60.0% (24/40) respectively at 2 months. This trend increased linearly over time until the success rate plateau at the end of fifth month and slightly decreased at the end of sixth month. The linearity of quitting rates in relation to time is illustrated in Figure 2.

Furthermore, at 3 months after the quit date, patients who received the integrated intervention had a significantly higher rate of success in quitting smoking when compared with those who received the usual TB care alone (70.0% vs. 10.9%, respectively; (Pearson χ^2 ^= 31.63, df = 1, N = 86; p < 0.001) (Table 5). In addition, at the end of the 6-month follow-up, the one-month self-reported CA rate, confirmed biochemically by both CO and saliva cotinine tests, was nearly 78% (31/40) in the intervention group versus 9% (4/46) in the usual care group (Pearson χ^2 ^= 41.97, df = 1, N = 86; p < 0.001).

**Table 5:** Smoking cessation outcomes according to group assignment

Outcome measure	DOTS (n = 46)	SCIDOTS (n = 40)	p-value^a^
	
	Abstinence rate, no. (%)		
**On quit date (Week 0)^b^**			
7-day point prevalence abstinence	0 (0%)	0 (0%)	**-**
Continuous abstinence (2 weeks)	0 (0%)	0 (0%)	**-**
**End of 3^rd ^month post quit date (Week 12)^b^**			
7-day point prevalence abstinence	6 (13.0%)	30 (75.0%)	< 0.001
Continuous abstinence (2 weeks)	5 (10.9%)	28 (70.0%)	< 0.001
**End of 6^th ^month post quit date (Week 24)^c^**			
7-day point prevalence abstinence	5 (10.9%)	33 (82.5%)	< 0.001
Continuous abstinence (4 weeks)	4 (8.7%)	31 (77.5%)	< 0.001

**^a^**Chi-square (χ^2^) test was used in calculating p-values.

**^b^**Patient-reported abstinence verified biochemically using breath CO analysis.

**^c^**Patient-reported abstinence verified biochemically using breath CO analysis and saliva cotinine testing.

### TB Treatment and Outcomes Monitoring

There were no significant differences between the two treatment groups in the distribution of anti-TB drugs regimen, duration of treatment, and drugs doses during both the intensive and the maintenance phases of treatment. Nearly all patients in both groups received the EHRZ regimen consisting of ethambutol, isoniazid, rifampicin, and pyrazinamide once daily dosing during the 2-month intensive phase, whereas more than 70% were on H_2_R_2 _regimen comprising of isoniazid and rifampicin twice weekly dosing during the 4-month maintenance phase. The median duration of the intensive phase was 2 months in both groups.

Sputum direct smear positivity for acid fast bacilli (AFB) was significantly higher among the participants in the integrated intervention group than among those in the usual care group at baseline (i.e. during diagnosis); 92.5% vs. 73.9% (Pearson χ^2 ^= 5.13, df = 1, N = 86; p = 0.023). However, the proportion of patients with positive sputum smear in both groups drastically decreased at the end of two months and four months of TB treatment, with no significant differences between the treatment groups (Table 6). Conversely, at the end of six months of DOTS regimen, the SCIDOTS group had significantly higher sputum smear conversion (negative smear test result) than did the DOTS group (100.0% vs. 93.9%; Fisher's exact p = 0.043).

**Table 6:** Group comparison of treatment response: sputum direct smear

Sputum AFB direct smear, no. (%)		Positive	Negative
Baseline	**Comparison Group** (n = 46)	34 (73.9%)	12 (26.1%)
	
	**Intervention Group** (n = 40)	37 (92.5%)	3 (7.5%)
	
	p-value	0.023^a^	

2 months	**Comparison Group** (n = 37)	7 (18.9%)	30 (81.1%)
	
	**Intervention Group** (n = 32)	6 (18.8%)	26 (81.2%)
	
	p-value	0.999^a^	

4 months	**Comparison Group** (n = 33)	2 (6.1%)	31 (93.9%)
	
	**Intervention Group** (n = 31)	1 (3.2%)	30 (96.8%)
	
	p-value	0.713^b^	

6 months	**Comparison Group** (n = 33)	2 (6.1%)	31 (93.9%)
	
	**Intervention Group** (n = 35)	0 (0%)	35 (100.0%)
	
	p-value	0.043^b^	

^a^Chi-square (χ^2^) test and ^b^Fisher's exact test were applied in calculating p-values.

AFB = acid fast bacilli

The baseline radiological presentations of the patients were not significantly different between treatment arms. Nevertheless, as the treatment progressed over time, significant differences were noted in terms of changes in X-ray findings, with subjects in the integrated intervention group showing better response to treatment (Table 7). At six months, the response rate was better among the integrated intervention patients than the standard care patients; proportions of patients with no lesion were 67.5% vs. 34.8%, respectively (Mann Whitney-U = 588.00; p = 0.001).

**Table 7:** Group comparison of treatment response: radiological findings

Chest X-ray changes, no. (%)		No lesion	Minimal	Moderately advanced	Far advanced
Baseline	**Comparison Group** (n = 46)	1 (2.2%)	16 (34.8%)	23 (50.0%)	6 (13.0%)
	
	**Intervention Group** (n = 40)	0 (0%)	21 (52.5%)	15 (37.5%)	4 (10.0%)
	
	p-value/MW-U	0.212/788.5			

2 months	**Comparison Group** (n = 45)	1 (2.2%)	22 (48.9%)	19 (42.2%)	3 (6.7%)
	
	**Intervention Group** (n = 40)	7 (17.5%)	25 (62.5%)	7 (17.5%)	1 (2.5%)
	
	p-value/MW-U	0.003/614.5			

4 months	**Comparison Group** (n = 46)	6 (13.0%)	21 (45.7%)	18 (39.1%)	1 (2.2%)
	
	**Intervention Group** (n = 38)	13 (34.2%)	22 (57.9%)	3 (7.9%)	0 (0.0%)
	
	p-value/MW-U	< 0.001/510.0			

6 months	**Comparison Group** (n = 46)	16 (34.8%)	16 (34.8%)	14 (30.4%)	0 (0.0%)
	
	**Intervention Group** (n = 40)	27 (67.5%)	11 (27.5%)	2 (5.0%)	0 (0.0%)
	
	p-value/MW-U	0.001/588.0			

Mann Whitney-Utest was applied in calculating p-values. MW-U = Mann Whitney-U

### Effect of the Intervention on TB Treatment Outcomes

Treatment default rate during two months and four months of TB treatment was higher among the conventional DOTS group when compared with the SCIDOTS group, although the difference at two months failed to reach statistical significance (Table 8). At the end of six months, however, the default rate was significantly lower among the integrated intervention (SCIDOTS) group when compared with the usual care (DOTS) group (2.5% vs. 15.2%; Fisher's exact p = 0.031). In addition, the cure rate was significantly higher among the former than the latter group (62.5% vs. 34.8%; Fisher's exact p = 0.031).

**Table 8:** TB treatment outcomes during follow-up according to group assignment

Outcome	**Comparison Group**, DOTS only (n = 46)	Intervention Group, DOTS + SCI (n = 40)	p-value^a^
	
	no. (%)		
**End of 2 months**			
Treatment interrupted	4 (8.7%)	0 (0%)	0.120
Treatment in progress	42 (91.3%)	40 (100.0%)	
**End of 4 months**			
Treatment interrupted	7 (15.2%)	1 (2.5)	0.045
Treatment in progress	39 (84.8%)	39 (97.5%)	
**End of 6 months**			
Cured	16 (34.8%)	25 (62.5%)	0.031
Treatment completed	6 (13.0%)	3 (7.5%)	
Treatment interrupted	7 (15.2%)	1 (2.5%)	
Treatment failed	3 (6.5%)	0 (0%)	
Treatment in progress	14 (30.4%)	11 (27.5%)	
**Overall treatment outcome**			
Cured	24 (52.2%)	32 (80.0%)	0.019
Treatment completed	12 (26.1%)	7 (17.5%)	
Treatment interrupted	7 (15.2%)	1 (2.5%)	
Treatment failure	3 (6.5%)	0 (0%)	

**^a^**Fisher's exact test was applied in calculating p-values.

DOTS = directly observed therapy short-course; SCI = smoking cessation intervention

Because TB treatment was extended beyond six months for many patients, we followed them in order to re-assess the overall TB treatment outcomes. The DOTS group when compared with the SCIDOTS group still maintained higher treatment default (15.2% vs. 2.5%) and failure rates (6.5% vs. 0%) (Table 8). Overall, the cure plus treatment completion rate (success rate) was 97.5% in the integrated intervention group, as compared with 78.3% in the comparison group (Fisher's exact p = 0.019).

## Discussion

The present study has investigated the outcomes of an integrated tobacco cessation intervention in TB care and its impact on TB treatment outcomes. When carefully interpreted, the results of the SCIDOTS Project may provide an evidence of the short-term benefits of connecting tobacco cessation intervention with TB DOTS. The study employed non-equivalent comparison group design for a question that may be better approached using a RCT design due to some major considerations. First, the provision of treatment for tobacco use and dependence in clinical settings is solely based on patient's readiness to quit smoking or stage of change, owing to the addictive nature of the disease and facts documented by the existing treatment models [33,38,39]. Evidently, tobacco use treatment may potentially fail if provided to a patient in the contemplation stage of change. Therefore, in order to run a RCT successfully, it is imperative to use effective and practical research strategies [40]. Second, the provision of tobacco cessation intervention is a medical imperative [41]; therefore, there is ethical dilemma in randomizing patients to no treatment (control group) when they are in the "preparation stage" of behavior change and given the potential positive impacts of quitting smoking on TB treatment outcomes and future lung health.

At the end of six months post-quit date, the current study has documented a tremendous abstinence rate of 78% among the patients undergoing tobacco cessation intervention versus only 9% among the patients who did not receive the value-added intervention. It is interesting to note that the increase over time in biochemically-confirmed patient-reported abstinence was linear. The smoking cessation rate recorded at the end of six month post-quit date in this study was much higher than that reported in the general population, which centers around 10 to 33% [35,38,42]. This is probably because patients who are ill are more likely to be receptive to health messages and comply with the advice given by the healthcare provider [29]. Therefore, treating tobacco dependence during TB treatment is a teachable moment that has an important implication on success to quitting [4,29]. However, the study compared persons motivated to quit smoking in an intervention group to those contemplating quitting smoking in a comparison group; therefore, the observed difference in smoking cessation between the groups would likely not have been so great.

Unfortunately, data with which a direct comparison of the current findings can be made are scanty since much of the previous studies were surveys based on previous TB patients self-reports or some form of observational studies. In a pioneering work performed by El Sony and colleagues in Sudan, the investigators reported a 66% (165/252) abstinence rate at the end of TB treatment among those who were enrolled in a tobacco cessation program vs. 14.3% (6/42) in the control group [43]. One important limitation of the former study was lack of biochemical validation of abstinence. The study also did not statistically compare changes over time in smoking abstinence between the intervention and the control groups. The intensity of the tobacco cessation intervention used in the present study may account for the higher quit rates observed in the integrated intervention group when compared with other studies. Particularly, participants enrolled in the integrated intervention group had 11 follow-up visits for smoking cessation as well as the use of both CBT and NRT for the treatment of their tobacco dependence. Perhaps, these strategies have lent some strength to the current study.

Furthermore, three recent observational studies from Jogjakarta Province of Indonesia, the Indian state of Kerala, and Yangzhong and Wujin County in China, have highlighted smoking behaviors among former TB patients [17,44,45]. In essence, 67-70% of former TB patients admitted to receiving smoking cessation advice or messages from healthcare providers at some point during their treatment in TB settings [44,45]. A major distinction between the former studies and the current study is that the messages given were typically short non-specific cessation messages mostly at the time of diagnosis that were not necessarily followed up during subsequent interactions with the physician or health professional DOTS provider. It is recommended that health professionals caring for TB patients should be formally trained and encouraged to promote smoking cessation, and to customize the interventions to those suitable for TB patients (i.e. TB disease-specific messages and interventions) [25,28,29,34,45].

The majority (over 80%) of TB patients would quit smoking during or soon after diagnosis, but at least one-third of the sample surveyed relapsed during the six months of treatment [44,45]. These findings though not consistent with our study, revealed that the messages received from healthcare providers in TB clinics could play a significant role in aiding smoking cessation and that more success might have been recorded if proactive tobacco cessation interventions were provided. In contrast to our study in which the trend in smoking abstinence over time was positively linear in the integrated intervention group, Ng *et al*. and Pradeepkumar *et al*. studies have demonstrated a negative linear relationship between quit rates and time. This implies that success in quitting smoking among the patients surveyed declined over time during and after the course of TB treatment. A probable explanation for such a distinct contrast between the present study and others is that the intensive nature of a tobacco cessation program plays an important role in achieving and maintaining quitting with lower chances of relapse. Previous studies in the general population are also in support of this hypothesis [46]. Most importantly, even the quit rates documented by the two other group of researchers might have likely been over-estimated, since quitting was measured on the basis of self-reports from former TB patients, subjecting the findings to high propensity of recall bias and social desirability issues.

It was interesting that sputum smear conversion rate was consistently higher among the SCIDOTS group than the DOTS group, although this was not statistically significant at the end of two months and four months. This indicates a potential effect of tobacco cessation on sputum smear conversion. A group of investigators found that smokers and non-smokers converted with similar frequency to a negative sputum status at the end of two months (p = 0.065; OR, 0.47) [47]. However, they noted that smokers with far advanced radiographic abnormalities or with 3+ smear status, were found to have a significantly lower chance of an early smear conversion (p = 0.038 and p = 0.011, respectively). Similarly, Leung *et al*. found that there was no statistically significant difference in the smear conversion rate at two months between smokers and non-smokers (OR 0.89, p = 0.655) [48]. These two previous studies, however, failed to investigate further the influence of smoking on sputum smear conversion beyond two months of TB treatment. In the current study, we documented a significant difference in the sputum conversion rates between participants in the integrated intervention and those in the comparison groups at the end of sixth month; suggesting that smoking may still delay sputum conversion after two months of TB treatment. This observation points to the needs for further investigations to elucidate the influence of continued smoking behavior on smear conversion throughout the course of TB treatment.

The delayed sputum smear conversion observed among some subjects in the usual care group, who apparently continued to smoke, might be explained by the cascade of immunological activities associated with tobacco smoking in causing infection and its persistence [1,20,49]. Interestingly, most of the immunological abnormalities associated with smoking are reversible within six weeks of smoking cessation [20-23]. Probably this supports the finding that an overwhelming proportion of the participants who received the combined TB-tobacco intervention had converted by four to six months of treatment. In a nutshell, the current findings suggest that sputum smear conversion is probably faster among TB patients who quit smoking than those who do not. This reaffirms the clinical implications of incorporating tobacco cessation as part of therapeutic plans for patients newly diagnosed with TB disease.

Contrary to our findings, in the El Sony *et al*.'s study, none of the differences between the TB treatment outcomes of the intervention and control patients was statistically significant [43]. Perhaps this is the only study that is comparable on head-to-head basis to the current study. This is because, at present, it is the only published study that involves prospective tobacco cessation intervention among patients with TB. However, the findings from that study should be carefully interpreted in the light of the potential limitations associated with it and somewhat variable study methodology and procedures when compared with the current study. Although the investigators separately followed both subjects at the intervention and the control centers, yet substantial proportions of the patients were non-smokers; 20% and 64% currently not using tobacco among participants in the intervention and control centers, respectively. This provides a hypothetical reason why the overall TB treatment outcome, particularly cure/completion rate was apparently better in the latter group than the former. The authors emphasized that the primary goal of their study was to examine the feasibility of including tobacco cessation into TB services and not to test the hypothesis that the combination would produce better treatment outcomes. We also found that treatment failure was significantly more common among participants in the usual care group compared to the participants in the integrated intervention group. This finding is not surprising, with the evidence of reversibility of smoking-related immunological abnormalities within six weeks after cessation [20,22,23], emphasizing the potentials for short-term benefits in recent quitters. Therefore, persistent smokers are at greater likelihood of persistent infectivity and poorer outcomes. Santha *et al*. found that TB patients under DOTS who currently smoked cigarettes were at a significantly higher risk of treatment failure than their counterparts who did not (OR 8.4, CI 1.0-388; p = 0.04) [11].

Several non-interventional studies have equally demonstrated the negative impact of tobacco smoking on TB treatment outcomes [8-11,17,50]. It has been previously documented that being a non-smoker is associated with a greater chance of adherence to TB treatment (OR = 1.8; 95% confidence interval, CI = 1.0-3.3) [19]. However, it is not well-known whether quitting smoking during TB treatment would have immediate impact and produce similar outcomes as those seen among never smokers. Wang and Shen found that 11.3%, 15.0%, and 19.5% of quitters, non-smokers, and persistent smokers were non-adherent to DOTS regimen respectively [17]. Upon adjustment for sociodemographic confounders, TB patients without smoking cessation were twice more likely to default treatment than those who achieved cessation (OR 2.03; 95% CI 0.99-4.18) [17].

Similarly, in the present study, the rate of default at the end of TB treatment was significantly higher among TB patients in the usual care group when compared with the patients in the integrated care group. This finding indicates that tobacco cessation intervention could influence patients' adherence to treatment during the course of DOTS. Of the seven patients who interrupted treatment in the usual care group, more than 50% did so by the end of intensive phase of treatment. Therefore, SCI should be initiated as soon as a patient is enrolled into a DOTS program [51]. Evidence on the direct effects of tobacco cessation on TB treatment outcomes is scanty, yet recent observational studies indicate that TB patients who continue to smoke during treatment remain at greater risk to default treatment [17-19]. These observations corroborate the current findings, which are in the context of an experimental environment.

In a Hong Kong study using a logistic risk model of default (predictive power 85%), Chang and colleagues found that the risk for defaulting treatment under DOTS could be accurately predicted by smoking (OR 3.0, 95% CI 1.41-6.39, p = 0.004) among other factors [10]. This is also supported by similar studies conducted in Tiruvallur District, South India and in Taiwan in which smoking among TB patients receiving DOTS was demonstrated to be significantly associated with a higher likelihood of treatment default (OR 2.1, CI 1.3-3.4; p < 0.01 and OR 2.45, CI 1.22-4.93; p < 0.05, respectively) [11,50]. From observations in SCIDOTS Project, we conclude that regular patient-provider interaction during DOTS reinforced by intensive tobacco cessation follow-ups may significantly improve adherence to TB regimens.

Overall, the present study underscores the potential benefits of smoking cessation in improving outcomes during the course of TB treatment. Therefore, the clinical and practice implications of the current findings include emphasis on the importance of connecting smoking cessation as part of the therapeutic plans for individuals newly diagnosed with TB.

There were a number of inevitable limitations in the present study. This study was a non-equivalent comparison group trial involving TB patients. The non-randomized approach to group assignment was in the light of the nature of nicotine addiction and ethical dilemma, which necessitated us to adopt the Transtheoretical Model of Stages of Change during the assignment of subjects into the treatment groups. Therefore, there were inherent potential threats to internal validity. One of these is selection bias, because assignment to groups was based on participant's decision. There was also relatively large dropout rate without intention-to-treat analysis. It remains possible that TB patients who participated may have differed in some important way from those who were lost to follow-up. In addition, the study was limited by its short follow-up periods; we did not follow subjects after completion of DOTS to determine maintenance of abstinence and long-term outcomes of TB. Furthermore, the difference in nicotine dependence between the intervention and the comparison groups might influence the study results considering that the latter group had higher nicotine dependence at baseline than the former. This baseline difference can be controlled by considering the baseline FTND score as a covariate in subsequent analysis. In such circumstance, analysis of covariance (ANCOVA) with repeated measures instead of analysis of variance (ANOVA) is a statistical strategy to control for such differences. Unfortunately, all the primary outcome variables were non-parametric, which precludes the application of this parametric test. Therefore, this was an important limitation to this study. It is also worthwhile to note that the success recorded in this study might be influenced by Hawthorne effect as the intervention group had follow-up visits more than the comparison group.

## Conclusions

The potential salutary effects of connecting smoking cessation to DOTS on improving TB therapeutic outcomes were confirmed by the present study. The findings suggest that the integrated approach may be beneficial and confer advantages on short-term outcomes and possibly on future lung health of TB patients who quit smoking. Whether these effects are transient or would be retained for a longer duration needs to be further investigated. The results of this study might have an important practice and policy implication in the revision of TB treatment guidelines globally.

## Competing interests

The authors declare that they have no competing interests.

## Authors' contributions

AA designed the study, developed its protocols, collected the data, performed the statistical analyses and interpretation of the data, and wrote the manuscript. MHNM conceived the research idea, participated in designing the study protocol, data analyses and manuscript writing. NMN, NAA, SASS, ARM, and AAM all contributed in the protocol development, data collection and analyses, and substantially helped in improving the intellectual contents and scientific merit of the entire manuscript. All the authors have read and approved the final version of the manuscript.

## Funding

This project was supported by a research grant provided by the Institute for Health Management, National Institutes of Health, Ministry of Health, Malaysia.
